# Femaleness for improving grain yield potential and hybrid production in barley

**DOI:** 10.1093/jxb/erad257

**Published:** 2023-09-12

**Authors:** Yongyu Huang, Thorsten Schnurbusch

**Affiliations:** Leibniz Institute of Plant Genetics and Crop Plant Research (IPK), Corrensstr. 3, OT Gatersleben, D-06466 Seeland, Germany; Leibniz Institute of Plant Genetics and Crop Plant Research (IPK), Corrensstr. 3, OT Gatersleben, D-06466 Seeland, Germany; Martin Luther University Halle-Wittenberg, Faculty of Natural Sciences III, Institute of Agricultural and Nutritional Sciences, D-06120 Halle, Germany

**Keywords:** Allocation, barley, carpel, grain yield, homeotic, sex determination, stamen

## Abstract

This article comments on:

Selva C, Yang X, Shirley NJ, Whitford R, Baumann U, Tucker MR. 2023. *HvSL1* and *HvMADS16* promote stamen identity to restrict multiple ovary formation in barley. Journal of Experimental Botany 74, 5039–5057.

**Homeotic genes have been known for decades to affect floral organ identity. Studying their functions in cereal crops is of particular importance because it is directly relevant for grain yield formation. Selva *et al*. (2023) identified two homeotic genes in barley whose mutations give rise to multiple ovary phenotypes due to homeotic conversions of stamens into carpels, one encodes a C2H2 zinc-finger protein (HvSL1) and the other encodes a B-class MADS-box protein (HvMADS16). Importantly, the supernumerary carpels in the *hvsl1* mutants remain fertile when cross-pollinated, representing a promising gateway for hybrid grain production and increased grain yield.**

## Insights from the ABCDE model

Flowers are key reproductive units for most plant species (angiosperms). They are typically composed of four whorls of floral organs sequentially arranged from the outside to the center of a flower, including sepals, petals, stamens (androecium), and carpels (gynoecium). Each type of floral organ has a distinct role during reproduction. For example, sepals can protect and support the inner floral organs; petals can attract pollinators (or humans); and stamens and carpels are male and female sex organs, respectively, which determine the ultimate reproduction. The spatiotemporal arrangements of these four floral organs are specified by the genetic hierarchy of certain groups of proteins referred to as the A-, B-, C-, D-, and E-class proteins according to the popular ABCDE model (Coen and Meyerowitz, 1991; Zahn *et al.,* 2006). Yet the proteins responsible for the identity of each floral organ are not simple homologous conversions among different plant species (Hirano *et al.,* 2014).

**Fig. 1.**
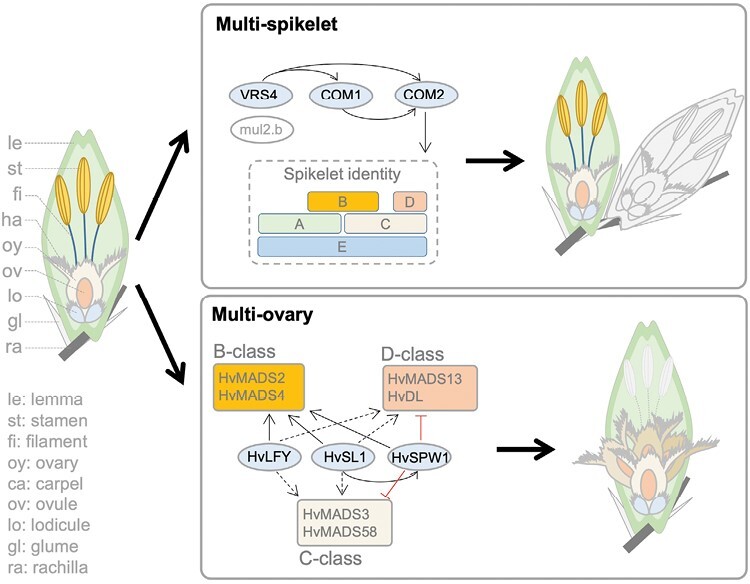
Two floral meristem identity/determinacy signals potentially leading to more grains in barley. A schematic representation of a wild-type spikelet morphology is shown on the left. Each floral organ is highlighted with different colors. Two proposed molecular pathways resulting in multispikelet or multiovary phenotypes are shown on the top right and bottom right, respectively. In barley, at least four loci have been shown to negatively affect spikelet number via the meristem identity/determinacy signals. Mutations in the *SIX-ROWED SPIKE4* (*VRS4*) gene encoding an ortholog of maize RAMOSA2 (RA2) results in a multispikelet phenotype due to the loss of spikelet determinacy (Koppolu *et al.,* 2013). *COMPOSITUM1* (*COM1*) and *COM2* encode TCP and AP2/ERF (ortholog of rice FRIZZY PANICLE) transcription factors, respectively, and determine inflorescence branching via the meristem identity signals (Poursarebani *et al.,* 2015, 2020). The *multiflorus2.b* (*mul2.b*) mutant also displayed a multispikelet phenotype, but the candidate gene is still not known (Koppolu *et al.,* 2022). In all cases, an extended outgrowth of the spikelet axis (rachilla) is observed, which potentially defines the extra spikelet formation. The regulatory relationships among the three cloned genes are based on Poursarebani *et al.* (2020). The ABCDE model is drawn according to Zahn *et al.* (2006). To improve visualization, the extra spikelet is shown in gray. The regulatory model for the formation of the multiovary phenotype is drawn according to Selva *et al.* (2021). Dashed arrows indicate insignificant or not major changes of the transcripts. Mutations in any of the three genes, namely *HvLFY*, *HvSL1*, and *HvSPW1 (HvMADS16*), all give rise to a multiovary phenotype due to the homeotic conversions of stamens into carpels. Only the extra ovaries from *hvlfy* and *hvsl1* mutants remain fertile, but not those in *hvspw1*. In *hvlfy* mutants, an incomplete conversion of stamens into carpels was observed; we therefore indicate the dispensable stamens with gray dashed lines.

In the *Triticeae* cereal crop barley (*Hordeum vulgare* L.), the sex floral organs contain three stamens (male part) and three fused carpels that enclose a single ovule (female part) (Fig. 1). The single ovary phenotype is maintained by at least three independent genetic loci termed *mov1*, *mov2*, and *mov5*; mutations in any of them give rise to a multiovary (*mov*) phenotype. Previous research by Selva *et al.* (2021) identified the barley *LEAFY* (*HvLFY*) homolog as the causal gene underlying the *mov5* locus. Now, Selva *et al*. (2023) focused on the *mov1* and *mov2* mutants, both of which showed homeotic conversions of the three stamens into additional carpels. In addition, lodicules (petal homologs) in the *mov1* mutant were converted into leaf-like structures but remained normal in the *mov2.g* mutant. Interestingly, ectopic carpels could be observed in the *mov2.g* mutant, but not in *mov1*, indicating that *MOV2* might also control carpel determinacy. Through map-based cloning and functional characterization, Selva *et al*. (2023) showed that the *mov2* locus was due to a deletion in the *STAMENLESS1* homolog (*HvSL1*) encoding a C2H2 zinc-finger protein, and the *mov1* locus was most probably due to a gene deletion in the B-class gene *MADS16* (*HvMADS16*), a barley homolog of rice *SUPERWOMAN1* (*SPW1*) and Arabidopsis *APETALA3* (*AP3*). They further showed that the *SL1*–*MADS16* regulatory module for the specification of stamen identity in rice (*Oryza sativa* L.) (Xiao *et al.,* 2009) was conserved in barley.

Some divergences, however, also existed. In rice, the *ossl1* mutant exhibited homeotic conversions of both lodicules and stamens into palea/lemma-like organs and carpels, resembling the *spw1*/*osmads16* mutant (Xiao *et al.,* 2009). Selva *et al*. (2023) clearly showed that lodicules were unaffected in the barley *mov2.g/hvsl1* mutant. Perhaps most striking are the additional carpels in *hvsl1* mutants that remain fertile when cross-pollinated, which was not known in rice. The work by Selva *et al*. (2023) thus not only provides insights into how floral architecture is controlled in a *Triticeae* crop—barley—but also demonstrates a promising way in which homeotic gene mutations can increase grain yield potential.

## The quest for more grains: a way forward

The broad implications for studying homeotic genes can be well demonstrated by the horticultural selection of many garden ornamental flowers such as roses and tulips (Bendahmane *et al.,* 2013). In these species, genetic manipulations on the ABCDE program may push the outward shift of the expression of B-class genes, thereby altering the organization of outward whorls of floral organs (e.g. sepals and petals) to provide aesthetic benefits for humans. In terms of plants for food purposes, such as many of the cereal crops, selections may tend to focus on the direction of more harvestable grains for human diets. Prominent examples are the genetic modifications of the meristem determinacy/identity signaling pathways leading to the increase of the final spikelet/floret number (Koppolu *et al.,* 2013, 2022; Poursarebani *et al.,* 2015, 2020; Dixon *et al.,* 2018), which often require a *de novo* assembly of the ABCDE program to establish the spikelet/floret identity (Fig. 1). An undesired trade-off commonly observed when more spikelets/florets are produced is the reduction in grain size or fertility because extra spikelets/florets would mean extra costs, among others the synthesis of new floral organs (e.g. lemmas and paleas) and vasculatures. This suggests that additional assimilation from the source side is required to achieve an improved source–sink balance. At a broader scale, the number of ovules per flower can span over several orders of magnitude among angiosperms (Burd *et al.,* 2009). In an evolutionary context, a female-biased allocation is more likely to emerge in self-pollinating hermaphrodites when flower size is increased (Charlesworth and Charlesworth, 1981). In this regard, the multiovary in one floret reported by Selva *et al*. (2023) appears to be a resource-conserving strategy for increasing grain number, if fertilization can be secured for barley. Intriguingly, a well-known yet unidentified dominant wheat (*Triticum aestivum* L.) mutant, called *Three Pistils* (*TP*) per floret (Peng, 2003), has already solved this fertility problem. The TP trait appears specifically interesting because it also offers a greater grain yield potential *per se*. Considering the prominent TP phenotype, it is quite likely that genes involved in ovule initiation and development, such as the B_Sister_-class genes *MADS29*, *MADS30*, and *MADS31* (Callens *et al.,* 2018; Wilkinson, 2019), may be affected in this mutant. By aligning the known genomic locations of all MADS-box genes from hexaploid wheat (Schilling *et al.,* 2020) with the TP mapping locus on the long arm of chromosome 2D (Peng *et al.,* 2008), only *MADS31*/*TaBS-D2* remains as the best candidate gene. In any case, only future work will be able to show whether this postulated candidate gene can be confirmed.

The male-to-female sex transition in the *mov2.g* mutant may raise another layer of research interest for hybrid breeding. Barley is a strictly self-pollinating crop that would require the generation of male-sterile plants for hybrid production. However, available approaches such as cytoplasmic male sterility (CMS) and chemical gametocides remain a major challenge, in particular for temperate cereals crops such as barley (Fernández-Gómez *et al.,* 2020). For floral sex determination, homeotic genes, especially those from the B-, C-, and D-class and their upstream regulators, have been studied in a considerable number of plant species (Feng *et al.,* 2020) and are continuing to emerge in less exploited crop species such as barley. Genetic approaches such as genome editing may enable a targeted cessation (or conversion) of male or female floral organ development at an unprecedented precision, thereby maximizing cross-pollination efficiency and ultimately grain set in an F_1_ grain production context. The study by Selva *et al*. (2023) therefore demonstrates a ‘kill two birds with one stone’ approach whereby mutations of homeotic genes can simultaneously promote outbreeding efficiency and capture the benefits from heterotic yield potential and stability.
